# Hyperpolarized ^13^C-glucose magnetic resonance highlights reduced aerobic glycolysis in vivo in infiltrative glioblastoma

**DOI:** 10.1038/s41598-021-85339-7

**Published:** 2021-03-11

**Authors:** Mor Mishkovsky, Olga Gusyatiner, Bernard Lanz, Cristina Cudalbu, Irene Vassallo, Marie-France Hamou, Jocelyne Bloch, Arnaud Comment, Rolf Gruetter, Monika E. Hegi

**Affiliations:** 1 Laboratory of Functional and Metabolic Imaging, École Polytechnique Fédérale de Lausanne (EPFL), Lausanne, Switzerland; 2 Neuroscience Research Center, Lausanne University Hospital (CHUV) and University of Lausanne (UNIL), Lausanne, Switzerland; 3 Service of Neurosurgery Lausanne, Lausanne University Hospital (CHUV) and University of Lausanne (UNIL), Lausanne, Switzerland; 4 Center for Biomedical Imaging (CIBM), École Polytechnique Fédérale de Lausanne (EPFL), Lausanne, Switzerland; 5 General Electric Healthcare, Chalfont St Giles, Buckinghamshire, HP8 4SP UK; 6 Department of Radiology, University of Geneva (UNIGE), Geneva, Switzerland; 7 Department of Radiology, University of Lausanne (UNIL), Lausanne, Switzerland

**Keywords:** Biophysics, Cancer, Chemistry, Biochemistry, Physical chemistry, Diseases, Cancer, Biochemistry, Biophysical chemistry

## Abstract

Glioblastoma (GBM) is the most aggressive brain tumor type in adults. GBM is heterogeneous, with a compact core lesion surrounded by an invasive tumor front. This front is highly relevant for tumor recurrence but is generally non-detectable using standard imaging techniques. Recent studies demonstrated distinct metabolic profiles of the invasive phenotype in GBM. Magnetic resonance (MR) of hyperpolarized ^13^C-labeled probes is a rapidly advancing field that provides real-time metabolic information. Here, we applied hyperpolarized ^13^C-glucose MR to mouse GBM models. Compared to controls, the amount of lactate produced from hyperpolarized glucose was higher in the compact GBM model, consistent with the accepted “Warburg effect”. However, the opposite response was observed in models reflecting the invasive zone, with less lactate produced than in controls, implying a reduction in aerobic glycolysis. These striking differences could be used to map the metabolic heterogeneity in GBM and to visualize the infiltrative front of GBM.

## Introduction

Glioblastoma (GBM) is the most common malignant primary brain tumor in adults, notorious for its resistance to multimodal therapy with a median survival of less than 2 years^1^. GBMs are characterized by genetic and morphologic intra-tumoral heterogeneity^2^, and consist of a core lesion—often with central necrosis—surrounded by an invasive tumor front that is pivotal for tumor recurrence^3^. Within the standardized recommendations for imaging brain tumors^4^, the central mass can be detected by T_2_-weighted magnetic resonance imaging (MRI) and is highlighted in post-contrast T_1_-weighted image^5^. The infiltrative front, characterized by a low density of invading cells that have migrated into a region with an intact blood–brain barrier (BBB), is in contrast usually indiscernible with these standard MRI techniques. Consequently, part of it may lie outside of the resected and treated area following surgery and radiation therapy.

Metabolic imaging techniques can offer additional and essential information for the assessment of tumoral tissue^6^. Because upregulated glycolysis and the associated increase in lactate production (Warburg Effect) is considered a hallmark of cancer^7,8^, glucose is the substrate of choice for metabolic imaging. Various techniques are available to study in vivo glucose metabolism non-invasively, giving complementary information on the metabolic pathways of glucose. The widely employed positron emission tomography (PET) of ^18^F labeled fluorodeoxyglucose (^18^F-FDG)^9,10^ is limited to detect the total glucose consumption, without distinguishing between the tracer and its phosphorylated form, and cannot directly inform on the metabolic pathways subsequent to glucose phosphorylation. Moreover, the physiologic glucose consumption in the normal brain generates a high background uptake of ^18^F-FDG, which is generally high in the gray matter, and moderate to high in the white matter, thus limiting its application for the detection of brain tumors^11^. Magnetic resonance spectroscopy (MRS) of non-hyperpolarized (i.e. thermally polarized) metabolites allows to study brain tumor metabolism non-invasively and with non-ionizing radiation^12,13^. Metabolite concentrations that were quantified from ^1^H MRS spectra were found to be changing in response to treatment and the metrics describing these characteristics are associated with survival. Moreover, metabolic abnormalities can be detected in the normal-appearing brain regions, emphasizing the potential of molecular imaging to visualize the infiltrative front^14,15^. Nonetheless, despite recent developments, the applicability of these techniques with clinical diagnostics is still challenging. Good quality ^1^H MRS spectrum requires high magnetic field homogeneity, and the measurement of the steady-state metabolite concentrations provides no direct information on metabolic fluxes. The dynamic measurements of non-hyperpolarized (i.e. thermally polarized) ^13^C MRS can bring kinetic information, but its low sensitivity necessitates long acquisition times and long infusion times.

MR detection of hyperpolarized (HP) endogenous compounds, on the other hand, using dissolution dynamic nuclear polarization (dDNP)^16^ can provide real-time metabolic information related to enzymatic activity^17^. The use of HP substrates has given important insights into GBM metabolism^18–21^. In particular, MRS imaging of hyperpolarized [1-^13^C] pyruvate has been shown as a promising technique to monitor metabolism in various clinical studies^22,23^ including brain tumor patients^24–26^. HP pyruvate experiments are limited to probe a specific portion of the tumor glucose metabolism. The pyruvate bypasses the glucose transporters by entering the cell via the monocarboxylate transporters (MCTs) and then exchanges its ^13^C label with the endogenous lactate pools^27^, providing a tool for lactate topography^22^. A recent metabolic study in a model representing the infiltrative front of GBM indicated a reduction in glucose uptake in the infiltrative GBM model as determined by PET, while the metabolic rate of glucose oxidation (CMRglc) quantified by non-hyperpolarized ^13^C MRS was not significantly reduced^28^. Hence, direct detection of tumor glycolysis may provide new insights.

The possibility to directly monitor tumor glycolysis using hyperpolarized ^13^C labeled glucose has been demonstrated in a lymphoma mouse model in vivo^29^. We have recently reported the real-time detection of cerebral de novo synthesis of [1-^13^C]lactate following the infusion of HP [^2^H_7_,^13^C_6_]d-glucose in mice^30^. Therefore, the aim of this study was to demonstrate the feasibility to monitor real-time metabolism of hyperpolarized [^2^H_7_,^13^C_6_]d-glucose in GBM mouse models and to employ this technique to further investigate the metabolic fate of glucose in the infiltrative zone of GBM of patient-derived xenografts in mice. We aimed to address the question of whether changes in glucose metabolism can be characterized by measuring the de novo lactate production through glycolysis, and whether these can delineate differences between the invasive zone of GBM from the normal brain by real-time detection of glycolysis.

## Results

**Figure 1:**
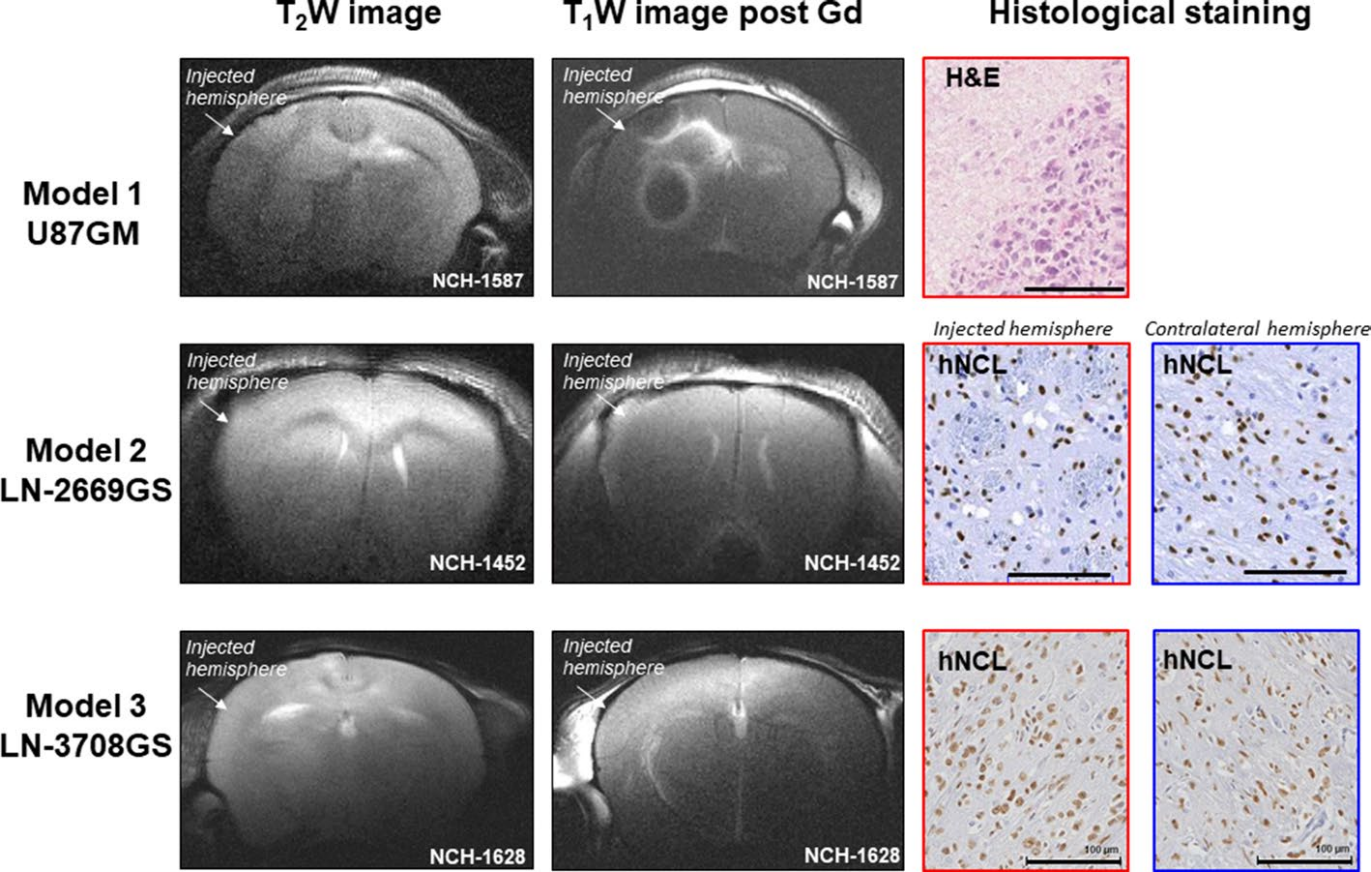
Axial slices of T_2_‐weighted images and T_1_-weighted images post injection of gadolinium contrast agent. The arrows indicate the injected hemisphere. As expected, implantation of U87GM cells lead to the development of a focal tumor as confirmed by hematoxylin and eosin (H&E) staining (model 1). Immunochemistry for human Nucleolin (hNCL) that detects human cells, visualizing the highly invasive properties in the injected and the contra‐lateral side in models 2 and 3, injected with LN-2669GS and LN-3708GS, respectively. NCH numbers indicate the identification number of each individual mouse.

Implantation of the U87GM cell line (model 1) yielded a compact tumor. A contrast-enhancing ring around a necrotic core can be readily observed in the post-contrast T_1_-weighted images, indicative of BBB disruption. Histology confirmed the observations of a compact tumor in the MRI images (Fig. 1). The patient-derived LN-2669GS (model 2) and LN-3708GS (model 3) sphere lines gave rise to diffusely invading tumors without visible modifications of the brain structure, and were characterized by lack of a focal mass, and absence of enhancement after gadolinium injection. Human tumor cells, visualized by immunostaining for human nucleolin, displayed highly invasive features, infiltrating also the contralateral hemisphere (Fig. 1).

### Metabolic profiling of tumor models

**Figure 2:**
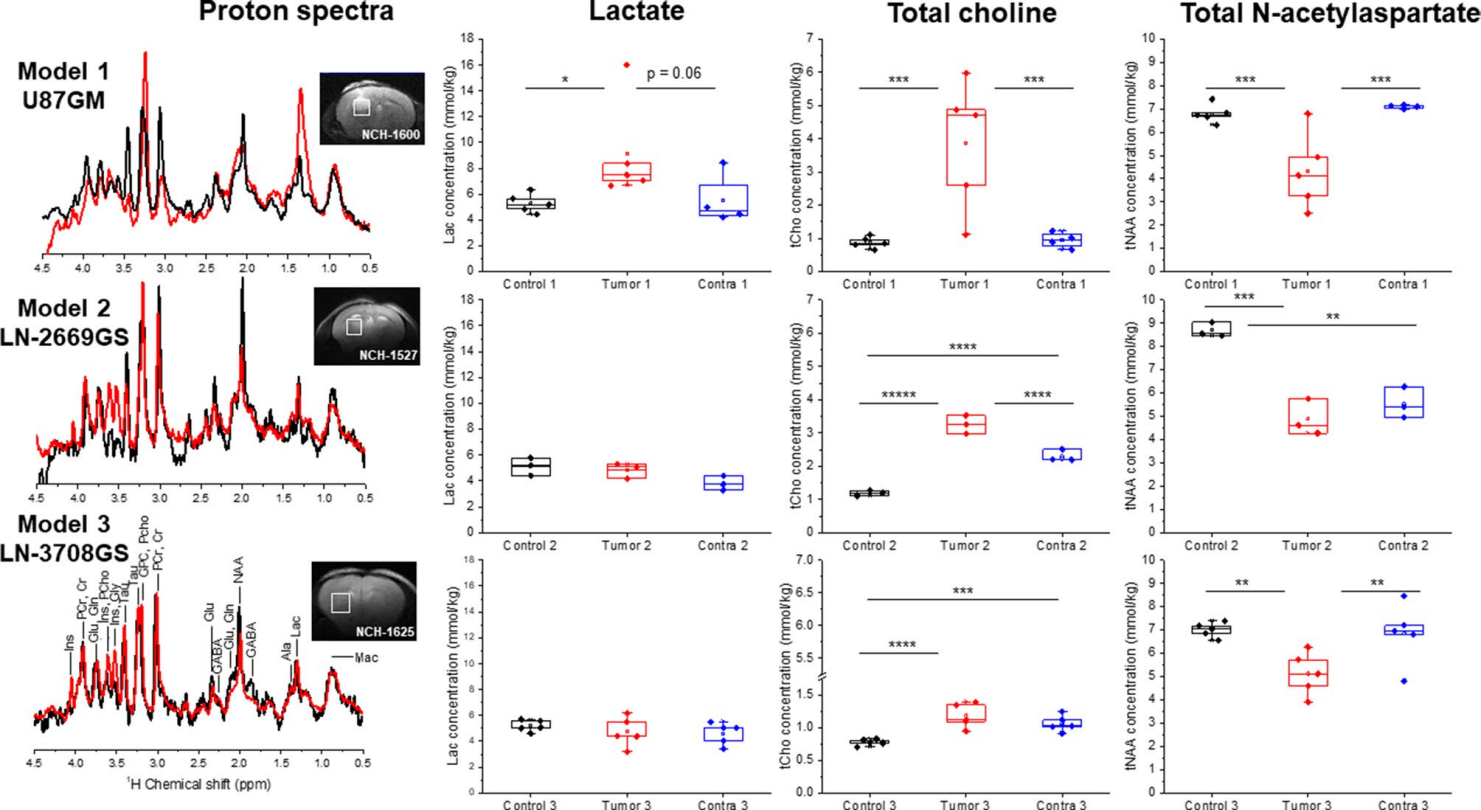
The steady-state concentrations of lactate, total choline (tCho) and total *N*-acetylaspartate (tNAA) concentrations at the day of HP glucose infusion. Localized ^1^H spectra were acquired in an 8 μL voxel, located in the cell injected hemisphere, as designated by the white square in the image (red spectra), corresponding voxel in the contralateral hemisphere (not shown) and in similar voxel in control animals (black spectra). The presented concentrations are only from the animals that received the HP ^13^C glucose bolus. In black are concentrations in the medium injected hemisphere in controls (control 1–3). In red, are concentrations in the tumor cell injected hemisphere (tumor 1–3), and in the blue concentrations in the contralateral hemisphere (contra 1–3) respectively, The number of ‘*’ represent statistically significant levels of p = 0.05, 0.01, 0.005, 0.001, 0.0005 and 0.00005. NCH numbers indicate the identification number of each individual mouse.

The metabolite concentrations derived from the ^1^H spectra resulted in distinct metabolic profiles of the tumor models as compared to the contralateral hemisphere and their corresponding controls (Fig. 2). The changes in cerebral metabolite concentrations enabled us to monitor the development of the infiltrative tumors post cell implantation and to sense cell migration to the contralateral hemisphere (Supporting Information Figure 1). In all three examined GBM models, the tumor tissue showed an increase in the concentration of total choline (tCho) metabolites that are related to membrane degradation and cell proliferation. The putative neuronal marker tNAA showed decreasing concentrations in all developing tumors of the injected hemispheres as well as in the contralateral hemispheres of the mice injected with the highly infiltrative model 2. The lactate pool size increased in model 1 in the area of the compact tumor mass but did not change in the infiltrative GBM models i.e. models 2 and 3 (Fig. 2).

### Real-time measurement of hyperpolarized glucose metabolism

**Figure 3:**
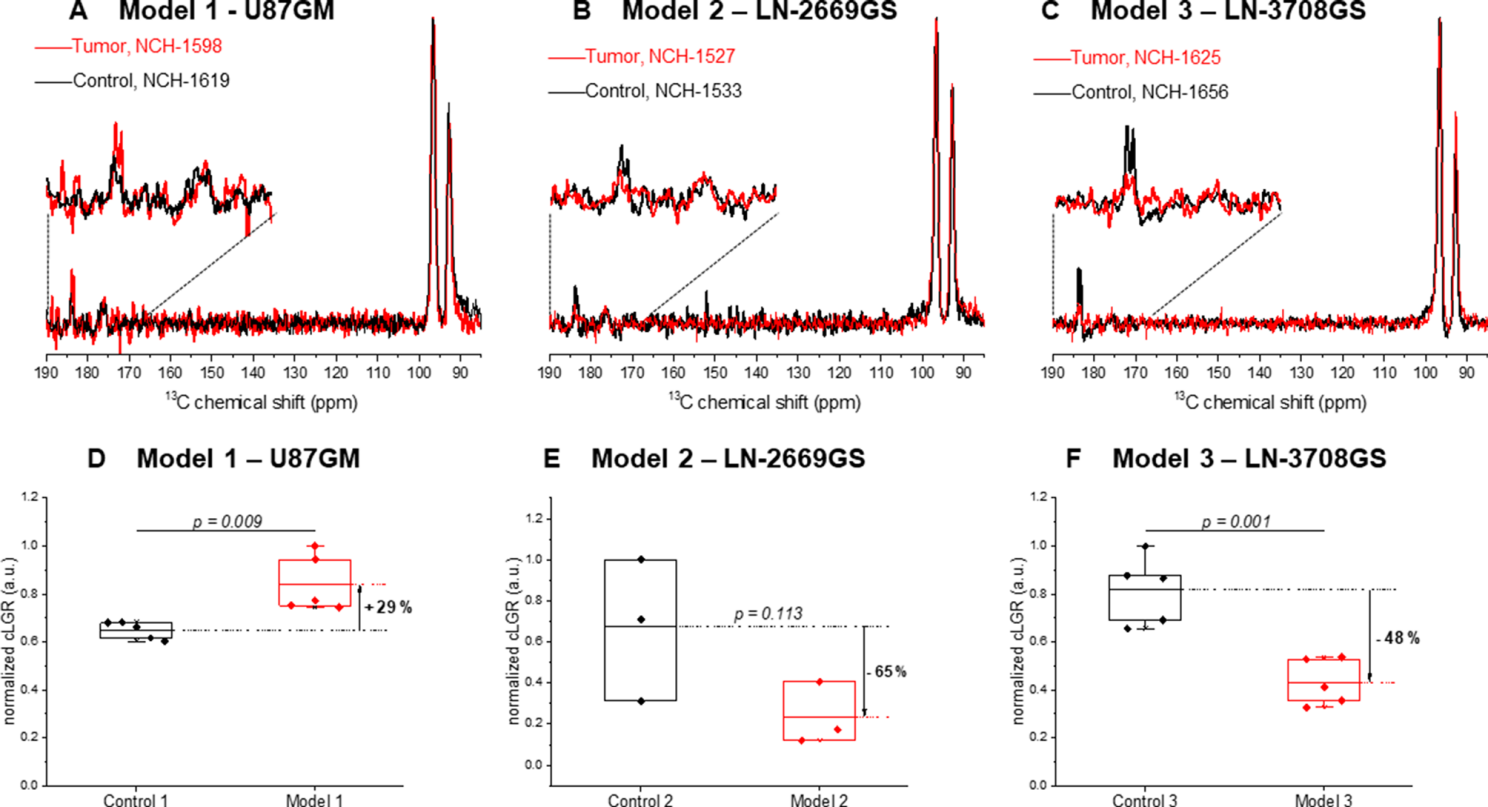
In vivo ^13^C magnetic resonance spectroscopy (MRS) of hyperpolarized glucose. (**A**–**C**) Characteristic summed spectra measured from the different tumor models over both hemispheres (red) and the corresponding controls (black) following a bolus of hyperpolarized (HP) [^2^H_7_,^13^C_6_] d-glucose (**A**–**C**). Spectra are normalized to their maximal glucose signal, respectively. In all acquisitions [1-^13^C] d-glucose-β (96.8 ppm), [1-^13^C] d-glucose-α (92.9 ppm) and [1-^13^C] Lactate (183.5 ppm) were detected. The broad peak at 175 ppm (designated by a star) is an impurity in the [^2^H_7_,^13^C_6_] d-glucose powder. (**D**–**F**) A comparison cLGR revealed an increase in the cLGR in model 1 compared to its control, and a decrease of the cLGR in infiltrative models (2 and 3) compared to the corresponding controls. cLGRs are normalized to the maximum cLGR observed in each pair to account for the differences in animals’ stains and age between the groups (Supporting Information Table 1). For all the measurements, we calculated blood glycaemia of 20.2 ± 3.6 mM and fractional enrichment of ^13^C glucose was 53.8 ± 10.2%, with no significant differences between the groups (Supporting Information Figure 3). NCH numbers indicate the identification number of each individual mouse.

The infusion of HP [^2^H_7_,^13^C_6_] glucose led to the production of [1-^13^C] lactate. The area under the curve (AUC) of [1-^13^C] lactate (183.5 ppm), [1-^13^C]α-glucose (92.9 ppm), and [1-^13^C]β-glucose (96.8 ppm) were quantified using VNMRJ software by integrating the sum of the ^13^C MRS spectra after phase and baseline correction. The lactate-to-glucose ratio (LGR) between the [1-^13^C] lactate signal and the sum of [1-^13^C] α- and β-glucose was calculated. The exact concentration of the glucose at the infusate solution was determined by high-resolution ^13^C NMR as previously proposed^31^. To account for the difference between animals and variances in infusate concentrations post dissolution, we multiplied those ratios by the actual concentration of the injected HP glucose divided by the animal blood volume, resulting in a corrected-LGR (cLGR) as previously described^32^. As anticipated, in the well characterized U87GM model (model 1) the [1-^13^C] lactate signals were higher in the tumor bearing brains compared to their controls, with the calculated the cLGR being 29% higher (p = 0.009) in the tumor bearing mice. Interestingly, an opposite trend was observed in the infiltrative GBM models 2 and 3, in which the signals of [1-^13^C] lactate after the HP ^13^C glucose bolus were smaller than in their corresponding healthy controls, respectively. The cLGR were reduced by 65% and 48% in the infiltrative models 2 and 3, respectively, as compared to their controls (Fig. 3). This difference was significant in model 3 (p = 0.001), however did not reach statistical significance in model 2 (p = 0.113). The latter may be due to the small number of mice, and the slightly lower ^13^C fractional enrichment of glucose in the control 2 animals (Supporting Information Figure 3). Note that blood glycemia and ^13^C glucose fractional enrichment that may influence these ratios were kept similar for all different measurements, and showed no significant differences between the groups (Supporting Information Figure 3).

## Discussion

Recent ex vivo and in vivo studies have shown evidences for distinct metabolic profiles associated with the invasive phenotype in GBM, indicating active glucose oxidation in human GBM^28,33^. In this work, we applied, for the first time, HP ^13^C labeled-glucose to further investigate glucose metabolism in GBM xenograft models in the mouse. The cLGR calculated from this measurement is connected to the rate of glucose uptake and real-time lactate production through glycolysis^29,30^. We examined three GBM models, two representing the infiltration zone (models 2 and 3), and are similar to the ones studied by Lai et al.^28^, and the U87GM model that leads to the formation of focal GBM mass (model 1). The latter is a well-characterized GBM model and was employed in this study to validate the reliability of the metabolic information detected by the cLGR from the HP glucose MRS. Namely, U87GM forms hypoxic tumors^34^, as such, hypoxia-induced factor 1 (HIF1) activates genes involved in extracellular glucose import and thus stimulating glycolytic energy production^35,36^. This was previously visualized by hyperintensity in ^18^F-FDG imaging^37^ and an increase in the endogenous lactate pool-size^34,38^ (Fig. 2). The HP ^13^C glucose MRS detection of a significantly higher cLGR in brains of U87GM mice (model 1) compared to control (control 1) was thus consistent (Fig. 3, + 29%, p = 0.009), and is in accordance with the metabolic shift anticipated by the so-called ‘Warburg Effect’^7,8^. Moreover, this higher labeling of lactate is consistent with previous studies with HP [1-^13^C]pyruvate in the U87GM tumor model that showed a high level of [1-^13^C]lactate labeling^39^.

Interestingly, in models 2 and 3, which reflect the infiltration zone of GBM, we found a different behavior of the cLGR. In model 3 the cLGR in the tumor bearing mice was significantly lower than in the corresponding controls (− 48%, p = 0.001), and in model 2 the same trend was observed (− 65%) without reaching statistical significance. Based on the cLGR values we found that de novo synthesis of lactate as detected by HP glucose MRS, was actually lower in the brains of mice implanted with the infiltrative models compared to their corresponding healthy controls. This indicates a reduction of aerobic glycolysis in infiltrative GBM, thus showing an opposite trend to what would be expected by the so-called ‘Warburg effect’. Previous work on patient-derived GBM models, has reported on a link between ^13^C labelling of lactate from a HP [1-^13^C] pyruvate bolus and c-Myc expression^40^. Upregulation of c-Myc may result in upregulation of GLUT1, MCT1, HK, and LDHA, thus one could anticipate a higher lactate labeling from both HP [1-^13^C] pyruvate and HP ^13^C glucose under c-Myc upregulation^41^. In light of that, lower lactate labelling from the HP glucose may be related to lower c-Myc expression in the infiltrative models studied in the present work. However further investigations are necessary to confirm this hypothesis. Moreover, in these tumors, the endogenous lactate pool size was similar to controls, in line with the absence of hypoxic conditions, as we have previously reported^28^. In addition, the labeling of [3-^13^C] lactate, after continuous infusion of thermally polarized [1,6-^13^C_2_] glucose, was similar in the infiltrative tumor model as compared to the control brain^28^. One could anticipate a similar response to a long infusion of thermally polarized [6,6′-^2^H_2_] glucose^42,43^. Thus the discrepancy between the lactate pool size and lactate labeling emphasizes the unique contrast one can detect with HP ^13^C glucose MRS, which enables monitoring the net ^13^C lactate production. It is likely that the reduction in the cLGR observed here, is a consequence of a decrease in the uptake of glucose. The present results support the work of Lai et al. on similar infiltrative GBM models, which indicated a reduction in glucose uptake as determined by ^18^F-FDG PET imaging, while the metabolic rate of glucose oxidation (CMRglc) quantified by ^13^C MRS was not significantly reduced^28^. Moreover, recent studies with HP pyruvate reported that although the lactate pool does play a role in the hyperpolarized lactate signal^27^, the pyruvate transport via MCT is of a major role in the detection of downstream metabolites post HP pyruvate bolus in some tumors^44–46^. A further step would be to examine perhaps a similar outcome might be possible with HP ^13^C-pyruvate in the infiltrative models investigated here. Of note, in the summed spectra of all the different models investigated in this study, we could only detect the resonances of the infused HP glucose and the produced lactate (Supporting Information Figure 4). Nonetheless, we cannot exclude the possibility that HP glucose was directed to the pentose phosphate pathway (PPP) to sustain proliferation by the production of amino acids, thus resulting in a reduction in the de novo synthesis of ^13^C labelled lactate. It is likely that the sensitivity of the current approach of using HP glucose can be further improved by optimal sample formulation^21^ or utilization of specifically labelled glucose^30^, and may lead to the observation of additional metabolites that could shed light on the metabolic pathway involved in the observed reduction in aerobic glycolysis.

The global acquisition strategy employed in this study probably led to an underestimation of the differences between the tumor metabolism of HP glucose in the brains implanted with U87GM tumor cells and respective mock injected control brains. The global detection scheme employed here confounds the possibility to define the metabolic response solely of the tumor tissue. The sensitive area under the coil likely was not only composed of tumor tissue (Supporting Information Figure 2). However, even using this somewhat limited detection mode, all measured parameters are coherent with what is anticipated by the Warburg effect in this well-characterized model. A localized detection scheme would indeed be preferable, and would enable discrimination between the tumor and the surrounding brain, and would eventually yield even more pronounced differences. For the infiltration models (models 2 and 3), the tumor cells diffused in the brain tissue (Fig. 1), hence the limitation in localization is inherent to these models, consequently, a localized detection would lead to a similar result to what we observed here by the global detection scheme.

Despite limitations of our acquisition strategy and the restricted number of animals, our results provide proof-of-principle evidence for the possibility of using HP [^2^H_7_,^13^C_6_] d-glucose to monitor lactate production in brain tumors. The need for novel techniques to visualize the invasive front of glioblastoma is well established. Here we demonstrate that the amount of freshly synthetized [1-^13^C] lactate produced after exogenous HP [^2^H_7_,^13^C_6_] d-glucose bolus is increased in the compact GBM tumor model, but decreased in the infiltrative GBM models as compared to their respective controls. Although technical challenges need to be overcome before introducing the cLGR in diagnostic imaging as a metabolic contrast and in particular limitations related to the short lifetime of the hyperpolarized state of glucose, it brings the potential to visualize the infiltrative front of GBM, displaying hyperintensity in the compact tumor part and hypointensity at the infiltrative front, a compartment highly relevant for tumor recurrence. Recent developments around photo-induced radicals have opened opportunities to reduce the delay between preparation and injection of hyperpolarized substrates, possibly allowing translation of hyperpolarized ^13^C-glucose^47–49^.

## Conclusion

We applied for the first time HP glucose MRS to study glucose metabolism in brain tumors, and found a reduction in aerobic glycolysis in a GBM model representing the infiltrative zone, in opposition to what is expected by the ‘Warburg effect’.

## Methods and materials

### Animal experimentation

Experiments were performed according to the Swiss law for the protection of animals and were approved by the Veterinary Office of the Canton de Vaud (Service de la consommation et des affaires vétérinaires, VD1181.6, VD2777, and VD3266). All experiments were conducted according to federal and local ethical laws and complied with the ARRIVE guidelines. Mice were given free access to food and water and were maintained in a 12 h light–dark cycle in a temperature- and humidity-controlled animal facility.

### Orthotopic mouse glioma xenograft models

To evaluate the metabolic performance of HP glucose MRS, the well-characterized U87GM GBM model was employed. U87 cells (10^5^), obtained from ATCC, were stereotactically injected into the left hemispheres of immunodeficient mice. This model will be referred to as model 1. To model the infiltrative compartment of GBM, the patient-derived glioblastoma sphere lines (GS) LN-2669GS (clone 822, n = 2; clone 867, n = 1)^50,51^ and LN-3708GS were stereotactically injected into the left hemispheres of immunodeficient mice as previously described (10^5^ cells in 5 μL Hank’s Balanced Salt Solution, HBSS)^52^, and will be referred to as Model 2 and 3, respectively. The GS lines were established in the laboratory with written consent of the patients (protocol F25/99, approved by the local ethics committee), and authenticated together with the original tumors using short tandem repeats (STR) (Dr. Vincent Castella, University Center of Legal Medicine, Lausanne, Switzerland). The corresponding controls (i.e. control 1, 2, and 3) were orthotopically injected with 5 μL of the cell suspension solution (HBSS) solely. All mice were 6 weeks old at the time of cell implantation. The age at the final HP ^13^C MRS measurement was dependent on the latency of tumor development and varied between the three models. Overall, 31 mice we employed in this study, among them 18 mice were injected with tumor cells and 13 mice were injected with the cell medium for control. Baseline information for the different groups is summarized in Supplementary Table 1.

### Hyperpolarization

HP of ^13^C-labelled glucose was prepared as previously described^30^. Briefly, [1,2,3,4,5,6,6-^2^H_7_, U-^13^C_6_]d-glucose (Sigma Aldrich) was dissolved in a polarization medium containing trityl radical OX63 (tris{8-carboxyl-2,2,6,6-benzo(1,2-d:5-d)-bis(1,3)dithiole-4-yl-methyl sodium salt) as a polarizing agent (ALBEDA, Denmark) dissolved in deionized water with 1:1.1 (w/w) ratio, to yield 3 M glucose and 25 mM OX63 radical solution. Frozen beads of the solution where dynamically polarized in a custom-designed 7 T DNP polarizer operating at 197 GHz/1.00 ± 0.05 K^53^ for 3 h, and then rapidly dissolved in 5 mL of superheated D_2_O and transferred within 2 s into the separator/infusion pump^54^, which was prepositioned inside the magnet bore. A bolus of the solution was then automatically infused through a vein catheter (see below) to the mouse as previously described^31^.

### Animal preparation for magnetic resonance scans

Mice were longitudinally scanned to monitor tumor development. Tumor bearing or control mice were anesthetized using 1.5 ± 0.5% isoflurane (Attane, Minrad, NY, USA) in 60% oxygen using a facemask. The anesthetized animals were then transferred to an MRI bed, and their head was fixed using a stereotaxic system and a bite bar (RAPID Biomedical Inc., OH, USA). Animal physiology was monitored during the entire duration of the experiment. Body temperature was monitored by a nonmagnetic rectal probe and maintained at 37.0 ± 0.5 °C by warming the animal with temperature-controlled water circulation (SA instruments Inc. NY, USA). The respiration rate was monitored using a pneumatic pillow sensor (SA Instruments Ins. Stony Brook, NY, USA).

Mice were selected for the HP glucose experiment when their physiology (i.e. weight loss), as well as MRI images and/or metabolites concentration from the MRS spectra indicated presence of a tumor (evolution of some metabolites in infiltrative models is presented in Supporting Information Figure 1). At the day of the HP glucose experiment, the anesthetized animals were cannulated. In experiments with models and controls 1 and 3, the catheter was positioned in the femoral vein. In experiments with model 2 and corresponding control 2, the tail vein was catheterized to deliver hyperpolarized glucose solution. After mice were transferred to the MRI bed, blood glycaemia levels were measured from blood samples (5 μL) collected from the tip of the tail (Breeze2 glucose meter, Bayer, France), and the animals was entered into the MR scanner.

All MR measurements were carried out on a Varian INOVA spectrometer (Agilent, Palo Alto, CA, USA) interfaced with a 31-cm horizontal-bore actively shielded 9.4 T magnet (Magnex Scientific, Abingdon, UK).

### In vivo ^1^H magnetic resonance imaging and spectroscopy

To confirm the presence of tumor we searched for morphological modifications (T_2_W images) and/or changes in the neurochemical profiles (localized ^1^H MRS). Spectra were acquired every second week starting 4 weeks post cell implantation. Measurements were performed using a home-built ^1^H-quadrature surface coil that was placed on top of the mouse head. T_2_W images were acquired using a fast spin echo multi slice (FSEMS) protocol (FOV 18 × 18 mm^2^, TR = 4000 ms, effective TE = 52 ms, 6 scans). B_0_ inhomogeneity was corrected using the FASTESTMAT algorithm^55^ in two 2 × 2 × 2 mm voxels, one located in the cell injection area, and the other one located in the corresponding area of the contralateral hemisphere. The positioning of the tumor voxel was guided by the location of the scar that was formed post cell implantation. Cells were injected at the same coordinates relative to the Bregma by means of a stereotactic frame, thus, this voxel is always located at the same general position. The contralateral voxel was taken as the mirror image of the tumor voxel. ^1^H MRS measurements were acquired using SPECIAL pulse sequences^56^ (TR = 4000 ms, TE = 2.8 ms, 200 ms acquisition time in 15 × 16 scans). Absolute metabolite concentrations were calculated using LC Model^57^.

The integrity of the blood–brain barrier (BBB) was assessed only on the day of the final experiment by T_1_ weighted (T_1_W) imaging post-injection of gadolinium contrast agent gadolinium (Gd3+) dihydroxy-hydroxymethylpropyl-tetraazacyclododecane-triacetic acid (Gadovist, Bayer Pharmaceuticals, France, 5 μL/g body weight). This procedure was done after the completion of the HP ^13^C MRS measurements, using FSEMS pulse-sequence (FOV 18 × 18 mm^2^, TR = 350 ms, effective TE = 11 ms, 6 averages).

### In vivo hyperpolarized ^13^C MRS

^13^C MR measurements were performed using a home-built ^1^H-quadrature/^13^C-single loop surface coil that was positioned on top of the mouse head. To improve the detection within the sensitive area of the ^13^C coil (Supporting Information Figure 2), B_0_ inhomogeneity was corrected using FASTESTMAP algorithm^55^ in a 135 μL voxel. A 540 μL of 59 ± 17 mM HP [^2^H_7_,^13^C_6_]d-glucose was injected through a vein catheter by the automated protocol^31^. A series of pulse acquired sequences was then triggered 5.5 s post injection every 0.5 s for 50 s. Frequency selective Gaussian pulse (250 μs) was centered at 182 ppm resulting an average nominal 20° flip-angle of the C1 lactate resonance (183.5 ppm) and 1.4° flip-angle on the glucose C1 resonances (92.9 ppm and 96.8 ppm) as previously proposed^30^. The area under the curve (AUC) of [1-^13^C] lactate (183.5 ppm), [1-^13^C]α-glucose (92.9 ppm), and [1-^13^C]β-glucose (96.8 ppm) were quantified using VNMRJ software by integrating the sum of the ^13^C MRS spectra after phase and baseline correction. Overall, 13 tumor mice, and 13 control mice received the HP glucose bolus (model 1, n = 5; control 1, n = 5; model 2, n = 3; control 2, n = 3; model 3, n = 5; control 3, n = 5).

### Histology and immunohistochemistry

Brains were fixed in formalin (4% buffered formalin), and embedded in paraffin. Coronal sections were stained by H&E (haematoxylin and eosin) or immunostained for human nucleolin (hNCL, 1:200, 4 °C, overnight; ab13541, Abcam, Cambridge, UK; does not react with mouse) to visualize the human GBM cells using a heat antigen retrieval procedure as previously described^58^.

### Statistical analysis

Statistical analyses were performed using the OriginPro 9.3G software. One-way analysis of variance (1-way ANOVA) was used followed by Fisher’s test to find significance in the difference between each tumor and its corresponding control. A *p* value of 0.05 was considered significant. All data are presented as means ± standard deviation unless otherwise stated.

## Supplementary Information

Supplementary Information.

## Data Availability

All data is available from the corresponding authors upon reasonable request.
