# Supplementary material for: Hyperpolarized 13C-glucose magnetic resonance highlights reduced aerobic glycolysis in vivo in infiltrative glioblastoma
Source: Sci Rep. 2021 Mar 11;11:5771. doi: 10.1038/s41598-021-85339-7 (PMC7952603; doi:10.1038/s41598-021-85339-7)
Supplement: Supplementary file 1 — Supplementary Information. [file 41598_2021_85339_MOESM1_ESM.docx]

Supporting information for manuscript:

**Hyperpolarized ^13^C-glucose magnetic resonance highlights reduced aerobic glycolysis in vivo in infiltrative glioblastoma**

Mor Mishkovsky^1*^, Olga Gusyatiner^2^, Bernard Lanz^1^, . Cristina Cudalbu^3^, Irene Vassallo^2^, Marie-France Hamou^2^, Jocelyne Bloch^2^, Arnaud Comment^4^, Rolf Gruetter^1,3,5,6^, and Monika E Hegi^2*^

**Supporting information Table 1:**

**Table 1:** Baseline information of animal specification included in this study.

| Group | Implanted cells/ HBSS* | Mouse strain | Total number of mice | Number of mice injected with HP* ^13^C glucose | Age at time of injection [weeks] | Weight at time of injection  [g] |
| --- | --- | --- | --- | --- | --- | --- |
| Model 1 | U87 | NSG^*^mice | 5 | 5 | 9 ± 1 | 25.7 ± 2.2 |
| Control 1 | HBSS^*^ | NSG^*^ mice | 5 | 5 | 9 ± 1 | 26.6 ± 1.9 |
| Model 2 | LN-2669GS | Swiss Nude mice | 8 | 3 | 22 ± 3 | 30.8 ± 0.8 |
| Control 2 | HBSS^*^ | Swiss Nude mice | 3 | 3 | 24 ± 2 | 32.0 ± 2.0 |
| Model 3 | LN-3708GS | NSG^*^ mice | 5 | 5 | 20 ± 1 | 31.4 ± 2.1 |
| Control 3 | HBSS^*^ | NSG^*^ mice | 5 | 5 | 20 ± 1 | 29.3 ± 1.8 |

*Abbrevations:

HBSS, Hank’s Balanced Salt Solution;

HP, hyperpolarized

NSG, NOD scid gamma (Jax name: NOD.Cg-Prkdcscid Il2rgtm1Wjl/SzJ)

**Supporting information Figure 1:**


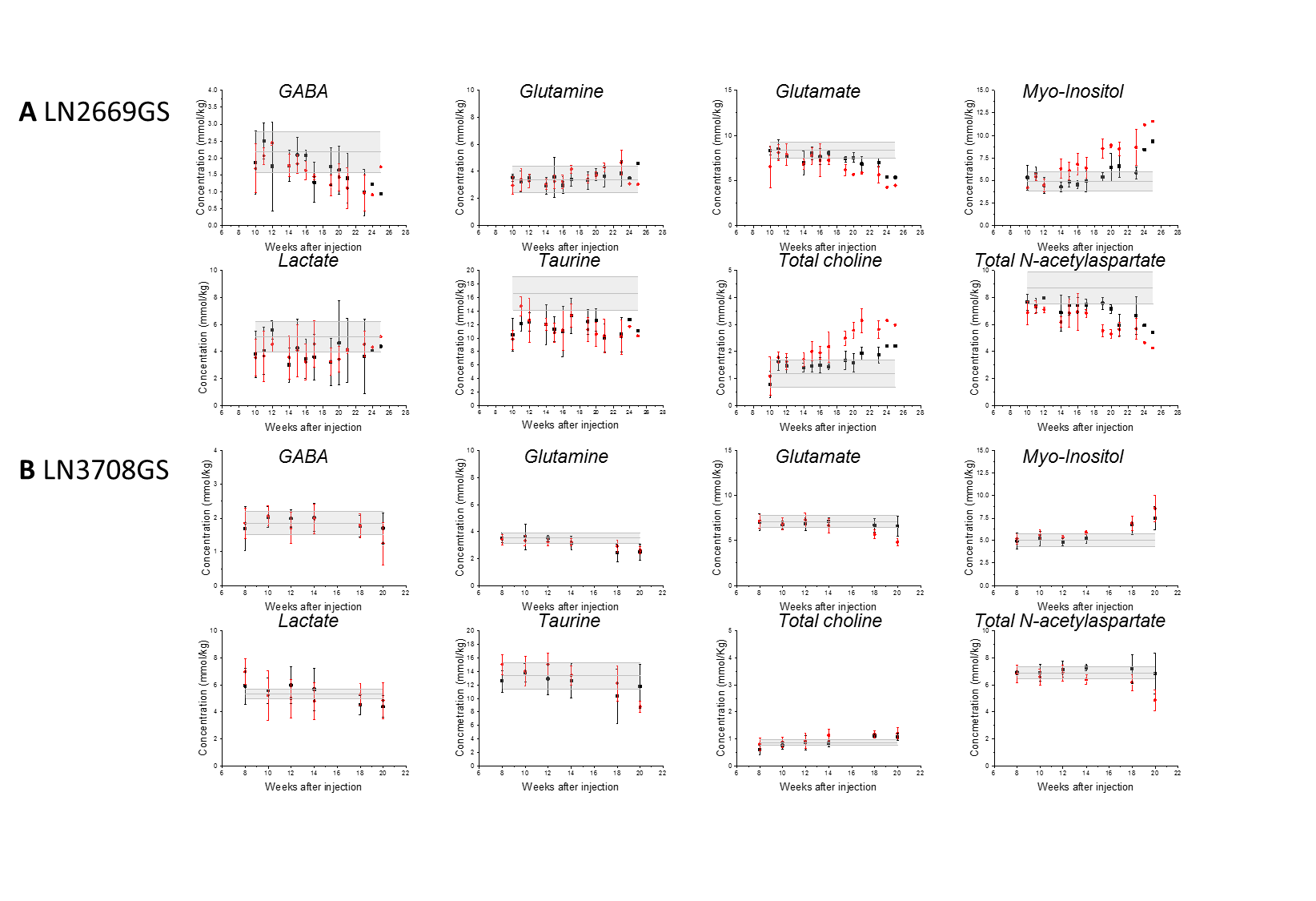


**Figure SI 1:** Absolute metabolite concentrations quantified from longitudinal ^1^H MRS measurements (mean ± SD). Two out of the seven LN2669GS mice (A) and five out of the seven LN3708GS mice (B) were used for HP glucose experiments. Red dots are the concentration measured in the injected hemisphere. Black dots are concentrations in the contralateral hemisphere. Grey bars indicates the concentrations detected in the injected hemisphere of sham operated mice at the last time point of the experiment. Of note, tumor cell invasion of the contralateral non-injected hemisphere can be monitored by measuring the changes in the metabolite concentrations.

**Supporting information Figure 2:**

**
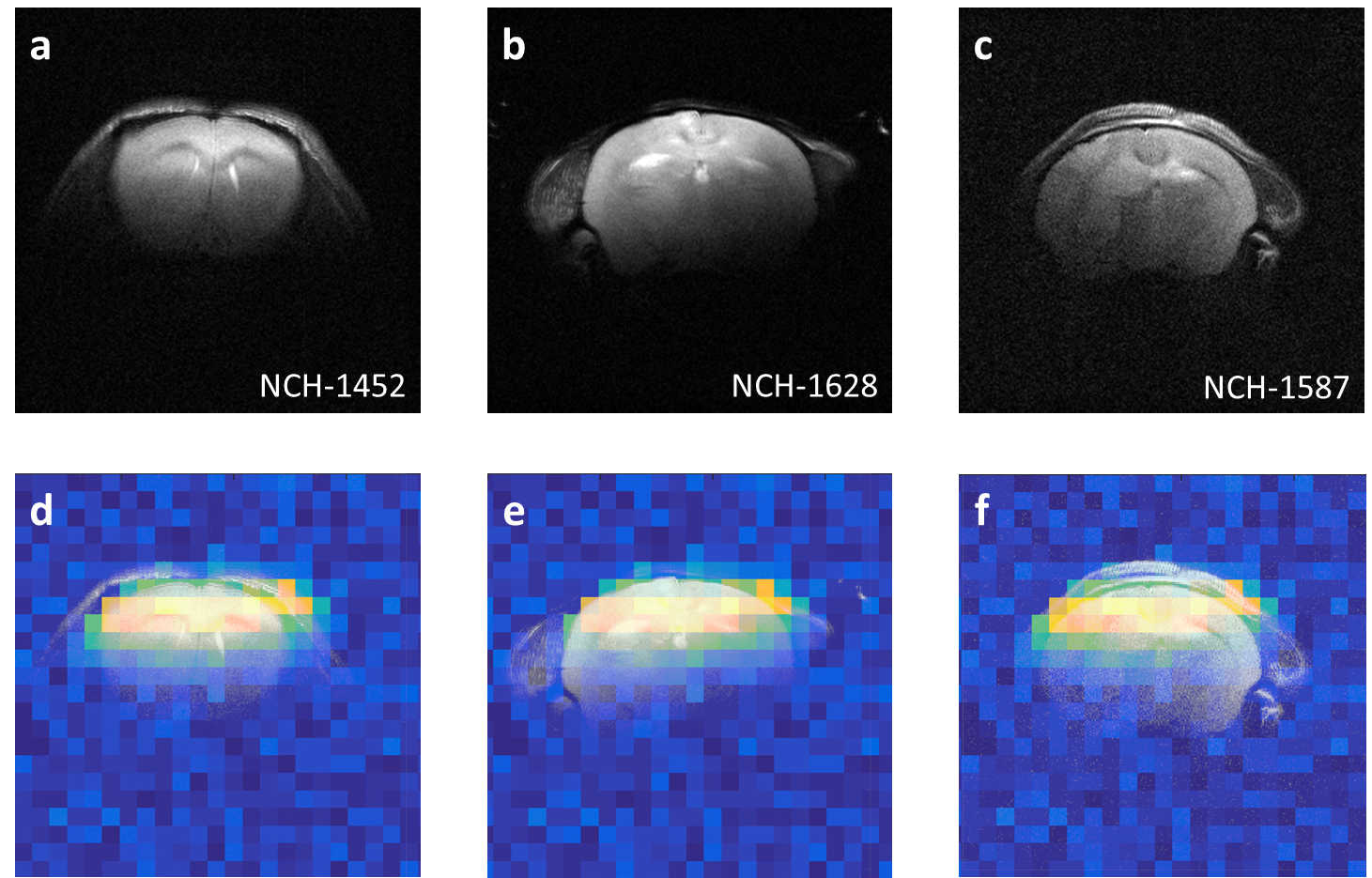
**

**Figure SI 2:** Anatomical T_2_W ^1^H image of LN-2669GS (a), LN-3708GS (b) and U87GM (c) mouse models and the overlay of the ^13^C B1+ map that is indicating the sensitive area of the coil on top of the anatomical images of LN-2669GS (d), LN-3708GS (e) and U87 (f) mouse models.

**Supporting information Figure 3:**


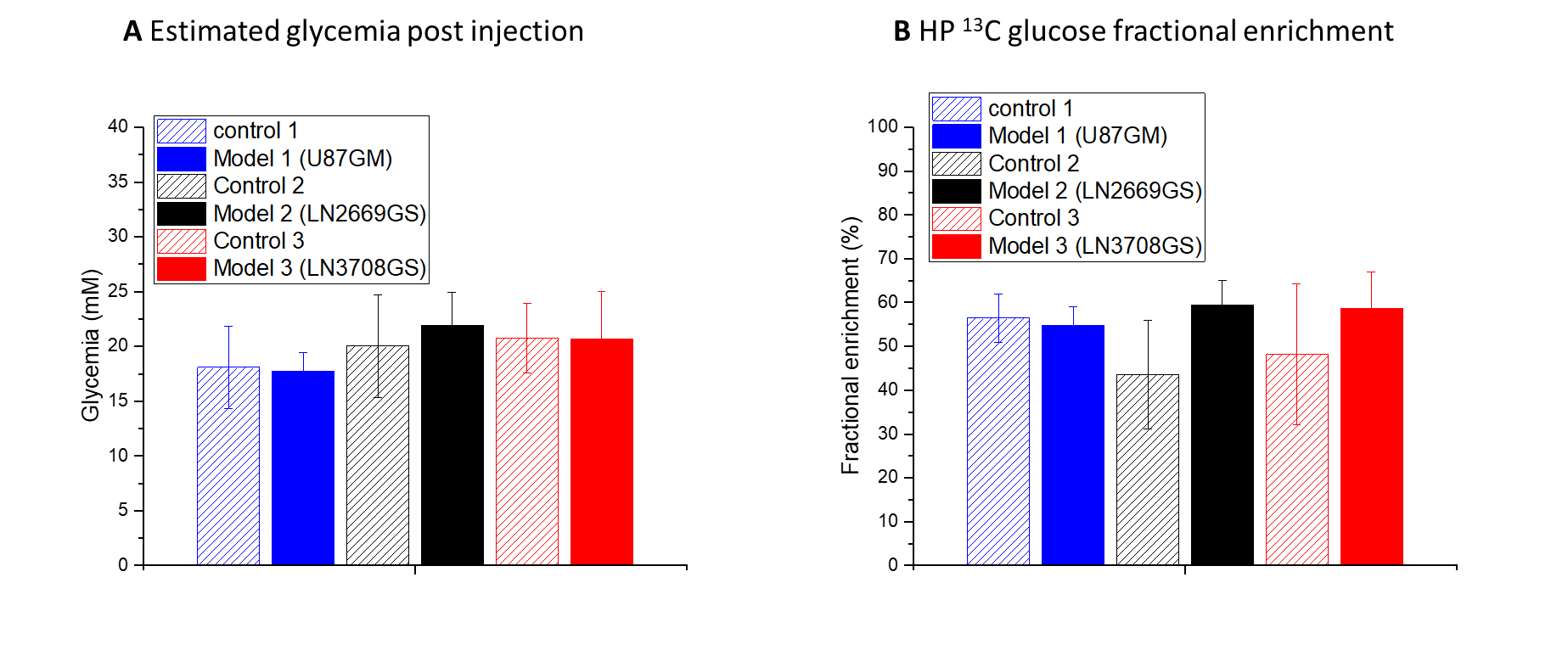


**Figure SI 3:** Estimated glycaemia levels and ^13^C glucose fractional enrichments. The glycaemia levels (A) post injection were calculated as the sum of the blood glycaemia before the bolus, as was measured by blood analysis, and the concentration of ^13^C labeled glucose in the infusate that was measured by HR ^13^C NMR. Fractional enrichment (B) was calculated as the ratio between the moles of ^13^C glucose in the infusate and total moles of glucose at the time of injection. No significant differences were found between the groups.

**Supporting information Figure 4:**


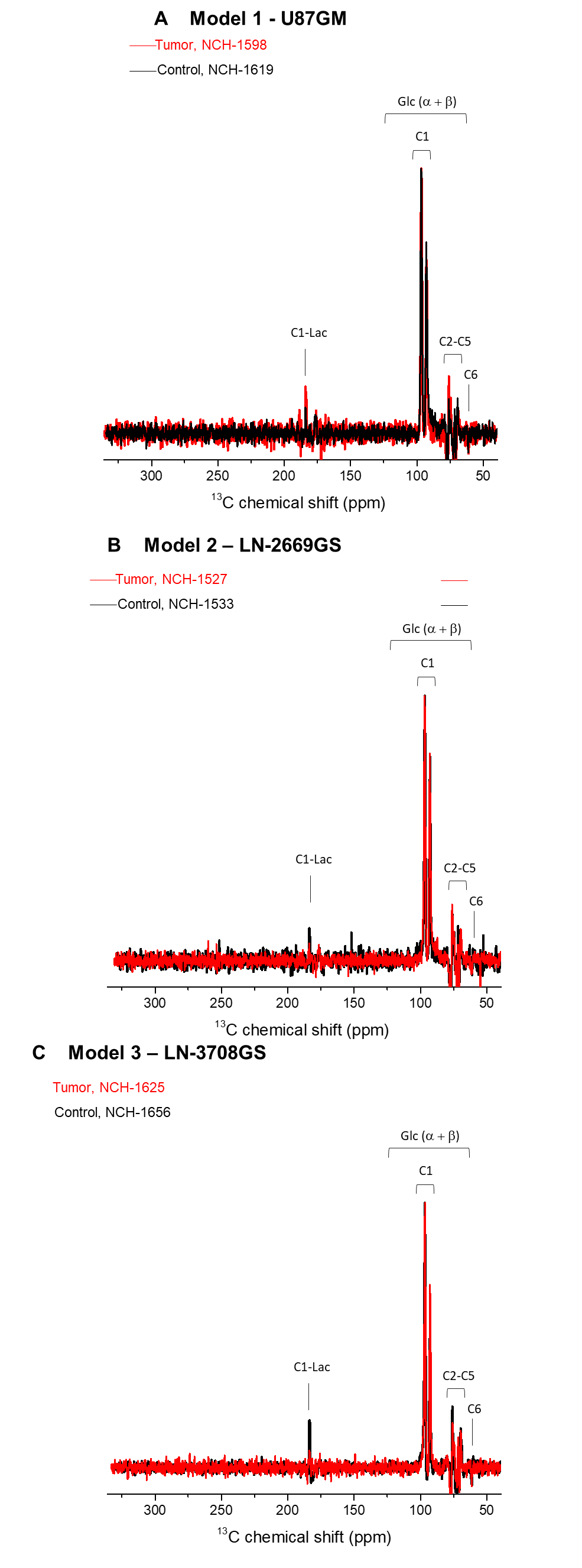


**Figure SI 4:** *In vivo* ^13^C magnetic resonance spectroscopy (MRS) of Hyperpolarized glucose. (A-C) Characteristic summed full spectra measured from the different tumor models over both hemispheres (red) and the corresponding controls (black) following a bolus of hyperpolarized (HP) [^2^H_7_,^13^C_6_] D-glucose (A-C). Spectra are normalized to their maximal glucose signal, respectively.
